# Attentional Disengagement and the Locus Coeruleus – Norepinephrine System in Children With Autism Spectrum Disorder

**DOI:** 10.3389/fnint.2021.716447

**Published:** 2021-08-31

**Authors:** Brandon Keehn, Girija Kadlaskar, Sophia Bergmann, Rebecca McNally Keehn, Alexander Francis

**Affiliations:** ^1^Department of Speech, Language, and Hearing Sciences, Purdue University, West Lafayette, IN, United States; ^2^Department of Psychological Sciences, Purdue University, West Lafayette, IN, United States; ^3^Department of Pediatrics, Indiana University School of Medicine, Indianapolis, IN, United States

**Keywords:** autism spectrum disorder, locus coeruleus, attention, disengagement, norepinephrine, pupil

## Abstract

**Background:**

Differences in non-social attentional functions have been identified as among the earliest features that distinguish infants later diagnosed with autism spectrum disorder (ASD), and may contribute to the emergence of core ASD symptoms. Specifically, slowed attentional disengagement and difficulty reorienting attention have been found across the lifespan in those at risk for, or diagnosed with, ASD. Additionally, the locus coeruleus-norepinephrine (LC-NE) system, which plays a critical role in arousal regulation and selective attention, has been shown to function atypically in ASD. While activity of the LC-NE system is associated with attentional disengagement and reorienting in typically developing (TD) individuals, it has not been determined whether atypical LC-NE activity relates to attentional disengagement impairments observed in ASD.

**Objective:**

To examine the relationship between resting pupil diameter (an indirect measure of tonic LC-NE activation) and attentional disengagement in children with ASD.

**Methods:**

Participants were 21 school-aged children with ASD and 20 age- and IQ-matched TD children. The study consisted of three separate experiments: a resting eye-tracking task and visual and auditory gap-overlap paradigms. For the resting eye-tracking task, pupil diameter was monitored while participants fixated a central crosshair. In the gap-overlap paradigms, participants were instructed to fixate on a central stimulus and then move their eyes to peripherally presented visual or auditory targets. Saccadic reaction times (SRT), percentage of no-shift trials, and disengagement efficiency were measured.

**Results:**

Children with ASD had significantly larger resting pupil size compared to their TD peers. The groups did not differ for overall SRT, nor were there differences in SRT for overlap and gap conditions between groups. However, the ASD group did evidence impairments in disengagement (larger step/gap effects, higher percentage of no-shift trials, and reduced disengagement efficiency) compared to their TD peers. Correlational analyses showed that slower, less efficient disengagement was associated with increased pupil diameter.

**Conclusion:**

Consistent with prior reports, children with ASD show significantly larger resting pupil diameter, indicative of atypically elevated tonic LC-NE activity. Associations between pupil size and measures of attentional disengagement suggest that atypically increased tonic activation of the LC-NE system may be associated with poorer attentional disengagement in children with ASD.

## Introduction

Differences in non-social attentional functions have been identified as among the earliest features that distinguish infants who develop autism spectrum disorder (ASD), and may play a critical role in the emergence of core ASD symptoms (Keehn et al., 2013). In particular, slowed attentional disengagement and difficulty reorienting attention (i.e., “sticky attention”) have been found across the lifespan in those at risk for, or diagnosed with, ASD (Sacrey et al., 2014). However, despite research highlighting these early emerging non-social attentional differences and their association with ASD symptomatology and later ASD diagnosis (Zwaigenbaum et al., 2005; Elison et al., 2013; Elsabbagh et al., 2013), the mechanism(s) underlying atypical attentional disengagement remain unknown.

To date, evidence of impaired attentional disengagement in ASD has been primarily demonstrated using gap-overlap paradigms, which have been employed from infancy through adulthood (see Sacrey et al., 2014, for review). Generally, these tasks examine differences in the latency of eye movements to peripheral targets, which appear with, or without, the presence of a central stimulus. Latency to execute saccadic eye movements (i.e., saccadic reaction time; SRT) is reduced when the fixated central stimulus is removed simultaneously with or prior to (e.g., 200 ms) the onset of a peripheral target compared to saccades generated when the central stimulus remains present (Saslow, 1967). The resulting difference in SRT is referred to as the gap (or step) effect, and is thought to result from two separate sources: (1) a generalized warning effect as a consequence of the central stimulus offset (i.e., an alerting cue), and (2) the release of ocular inhibition due to (a) the disappearance of a foveal stimulus, and (b) the top-down preparation of a saccadic response (Kingstone and Klein, 1993; Taylor et al., 1998). Although a large body of research has examined the neural circuitry associated with the generation of saccadic eye movements (see Liversedge et al., 2011, for example), a limited number of studies have investigated the neural substrates specifically associated with the gap effect.

Early evidence from studies investigating the neural mechanisms associated with latency differences between gap and overlap conditions has predominantly come from research with non-human primates. Unique patterns of gap-period activation in neurons of the superior colliculus have been linked to faster SRTs and increased frequency of express saccades [i.e., fast latency saccades (RT < 140 ms); Fischer and Weber, 1993) in the gap condition (Dorris and Munoz, 1995; Dorris et al., 1997)]. Other work has shown that neurons in the frontal eye fields (FEF) may increase their firing rate during the gap period (Dias and Bruce, 1994). Research investigating attentional disengagement in human adults with focal brain lesions has shown that increased saccadic latency for the overlap (but not the gap) condition is associated with lesions in both the frontal eye fields (FEF; Rivaud et al., 1994) and the posterior region of the anterior cingulate cortex (ACC; Gaymard et al., 1998). More recently, an fMRI investigation of gap-overlap performance in neurotypical adults showed that slower SRT for the overlap condition was associated with decreased activation in the bilateral inferior frontal junction (Ozyurt and Greenlee, 2011). These authors hypothesize that saccade generation while maintaining an active fixation on the central stimulus may require greater processing effort (especially compared to gap trials), and that efficient responding for overlap trials may require increased activation of this area, which is involved in task switching and set shifting. Together, these results suggest that the gap effect is generated by a combination of cortical and subcortical sources associated with condition-specific changes in SRT.

Although experimental parameters vary widely across previous studies (see Sacrey et al., 2014, for discussion), when ASD-related differences in task performance are present these tend to be exhibited as disproportionally longer SRT to *overlap trials* relative to gap trials compared to their typically developing (TD) peers. To date, only one study has examined the neurofunctional correlates of gap effect in ASD. Kawakubo et al. (2007) measured event-related potentials (ERP) during a gap-overlap task to investigate the neurophysiological indices of attentional disengagement. Compared to both TD adults and adults with intellectual disability, adults with ASD showed greater pre-saccadic positivity (PSP) for overlap but not gap trials. Prior work in neurotypical adults has shown that the PSP is greater for overlap compared to gap trials and may reflect greater activation of cortical eye-movement control network necessary to disinhibit the collicular system in overlap trials (Gomez et al., 1996; Csibra et al., 1997). Thus, larger PSP in ASD may reflect increased cortical activation necessary to initiate saccades in circumstances when individuals are engaged or fixating on a central stimulus (i.e., overlap trials).

Additionally, based on the pattern of prior gap-overlap findings in ASD, Keehn et al. (2019) hypothesized that disengagement impairments may reflect atypical activation of the locus coeruleus – norephinephrine (LC-NE) system. The LC-NE system is known to play a key role in arousal regulation and selective attention (Berridge and Waterhouse, 2003), and is an important node in two influential models of attention (Corbetta et al., 2008; Petersen and Posner, 2012). Norepinephrine functions to inhibit spontaneous neural activity, thereby permitting increased neural responses to sensory stimulation, thus increasing signal-to-noise, especially in sensory areas (Foote et al., 1975). Tonic (i.e., resting or baseline) activation of the LC-NE system is associated with regulation of the sleep-wake cycle, with lower tonic activation seen in sleep and greater tonic activity with increased arousal during the awake state; phasic activation of LC neurons occurs in response to salient or behaviorally relevant stimuli (Aston-Jones and Cohen, 2005). According to the adaptive gain model (Aston-Jones and Cohen, 2005), intermediate levels of tonic LC-NE activity are associated with robust phasic LC activation to task-relevant stimuli and superior task performance, whereas elevated tonic LC-NE activity is related to decreased phasic LC responses as well as poorer task performance and greater levels of distractibility.

Prior work has hypothesized that the LC-NE system may be implicated more generally in the pathophysiology of ASD (London, 2018) and more specifically related to differences in attention present in ASD (Bast et al., 2018). While previous findings based on plasma NE metabolites suggests that NE may be elevated in individuals with ASD (Lam et al., 2006), histological (Martchek et al., 2006) and positron emission tomography (PET; Kubota et al., 2020) findings suggest equivalent LC volume and cell counts as well as norepinephrine transport binding in ASD. However, the size and location of the LC (a small nucleus located adjacent to the fourth ventricle in the rostral pons) has made the study of LC activity difficult. More recently, pupil diameter has been shown to be an indirect index of LC-NE activity (see Joshi and Gold, 2020, for review). For example, several studies have shown that LC activity is associated with resting pupil size (Joshi et al., 2016; Reimer et al., 2016). Together, these results suggest that resting pupil diameter is valid proxy measurement for tonic LC-NE activity. However, only a limited number of studies (*n* = 5) have examined resting tonic pupil size in ASD (see Arora et al., 2021, for review). Of these, half have reported significantly increased pupil diameter in ASD, which may reflect increased tonic activity of the LC-NE system. Arora et al. (2021) note that measurement of autonomic indices (including pupil diameter) during resting state without stimulation (i.e., maintaining fixation to a central stimulus, not passively attending to multiple images or flashes) is more likely to produce significant group differences. For example, multiple studies that have recorded pupil diameter during extended periods of *rest* while maintaining fixation to a centrally presented crosshair have provided evidence of increased pupil diameter in ASD (Anderson and Colombo, 2009; Anderson et al., 2013; Top et al., 2018), whereas studies examining *baseline* pupil diameter within the context of stimulus presentation have not (de Vries et al., 2021). According to the adaptive gain model (Aston-Jones and Cohen, 2005), atypically increased resting, tonic LC-NE activity in individuals with ASD may be associated with reductions in phasic responsiveness; this may lead to slower, more variable, attentional disengagement, as the onset of peripheral targets does not result in robust phasic activity when attending to the fixation. For example, failure to make a saccade (i.e., a no-shift trial) and/or slower SRTs in the overlap condition may result from a more general inability to detect and respond to behaviorally relevant events (i.e., the target), especially when targets are not preceded by a cue (i.e., the offset of the fixation). Thus, increased tonic activation and associated reductions in phasic LC responsiveness may contribute to the presence of disengagement impairments in ASD.

The objectives of the present study were to investigate tonic LC-NE activity in ASD (as indexed by resting pupil diameter), and to test the hypothesis that elevated tonic LC-NE activity is associated with impairments in attentional disengagement in children with ASD. To examine this association, a resting pupillometry experiment was conducted in conjunction with two gap-overlap paradigms: one auditory (Keehn et al., 2019) and one visual. Additionally, given that ASD may be associated with impaired zooming out of attention (Mann and Walker, 2003; Ronconi et al., 2013, 2018), we examined disengagement and shifting attention to targets occurring at both near and far distances from the central fixation. Our previous report (Keehn et al., 2019) showed that deficits in auditory attentional disengagement are present in children with ASD; however, in the present study, we expand our investigation to focus on a potential mechanism related to auditory and visual attentional disengagement impairments in ASD – atypical LC-NE activation. We hypothesized that children with ASD would evidence atypically increased resting pupil size (indicative of greater tonic LC-NE activation) and impaired auditory and visual attentional disengagement (which would be greater for targets occurring at larger distances from the central fixation), and that elevated LC-NE activation would be associated with poorer attentional disengagement in ASD in both sensory modalities.

## Materials and Methods

### Participants

Twenty-one children with ASD and 20 age- and IQ-matched TD children participated in the study (see Table 1). The current manuscript includes the same participants from Keehn et al. (2019). Clinical diagnoses were confirmed using the Autism Diagnostic Observation Schedule, Second Edition (ADOS-2; Lord et al., 2012), Social Communication Questionnaire (SCQ; Rutter et al., 2003), and expert clinical judgment according to DSM-5 criteria. Children with ASD-related medical conditions (e.g., Fragile-X syndrome, tuberous sclerosis) were excluded. Participants in the TD group had no reported family history of ASD and were confirmed via parent report to be below clinical cutoffs on the Social Responsiveness Scale, Second Edition (Constantino and Gruber, 2012). Informed assent and consent were obtained from all participants and their caregivers in accordance with the Purdue University Institutional Review Board.

**TABLE 1:** Participant characteristics.

		ASD	TD	Statistic	*p*
n (M:F)		21 (17:4)	20 (15:5)	χ = 0.2	0.65
Age (years)		11.5 (1.3); 9.2–14.5	11.2 (1.5); 9.3–15.0	*t* = 0.6	0.57
Verbal IQ		102 (19); 69–154	110 (11); 95–127	*t* = -1.5	0.14
Non-verbal IQ		101 (20); 52–132	111 (13); 87–134	*t* = -1.9	0.07
SRS-2 total score		75 (11); 57–90	44 (5); 37–55	*t* = 11.6	<0.001
ADOS-2	Social affect	11 (4); 5–17	–	–	–
	Repetitive behavior	2 (1); 0–5	–	–	–

*IQ determined using the Wechsler Abbreviated Scale of Intelligence, Second Edition (WASI-II; Wechsler, 2011). Mean (SD); range.*

### Resting Pupil Dilation

#### Apparatus

The experiment was presented using SR Research Experiment Builder 2.1 on a 17-inch LCD monitor. Participants were seated approximately 60 cm from the display. Eye movements and pupil diameter were recorded (500Hz; monocular) using an EyeLink 1000 Plus remote eye-tracking system (SR Research, Ontario, Canada).

#### Procedure

Participants first completed a nine-point calibration and validation procedure. Next, participants completed a total of six minutes of eyes-open resting eye tracking (3, 2-minute blocks with breaks in between). A black central fixation was presented on a gray background, and participants were instructed to relax, look at the crosshair, and remain as still as possible. Background illumination of the room was fixed (450 lux).

#### Preprocessing and Analysis

A procedure similar to that described by Steiner and Barry (2011) was used to convert arbitrary units reported by Eyelink eye tracker to millimeters. Briefly, prior to data collection, an array of simulated pupils (2–10 mm) were placed at multiple distances (550–700 mm) from the eye tracker, using multiple thresholds. These measurements were entered into a multiple linear regression and coefficients from this analysis were used to predict absolute pupil size for the current data set.

Periods in which the eye tracker did not record pupil diameter were considered artifacts and excluded (e.g., blinks and saccades). Furthermore, instances in which the pupil size exceeded 1.5× interquartile range were considered outliers and were removed from the data. Lastly, data 200 ms before and after periods of missing or excluded data were removed. Missing and excluded data were corrected using linear interpolation.

In addition, the distance between the eye tracker and the forehead of the participant was used to monitor participant movement. Specifically, the root mean square of the first temporal derivative of the distance measurement was used a metric of overall head movement during resting pupil recording.

### Visual Gap-Overlap Experiment

#### Apparatus and Stimuli

**FIGURE 1:**
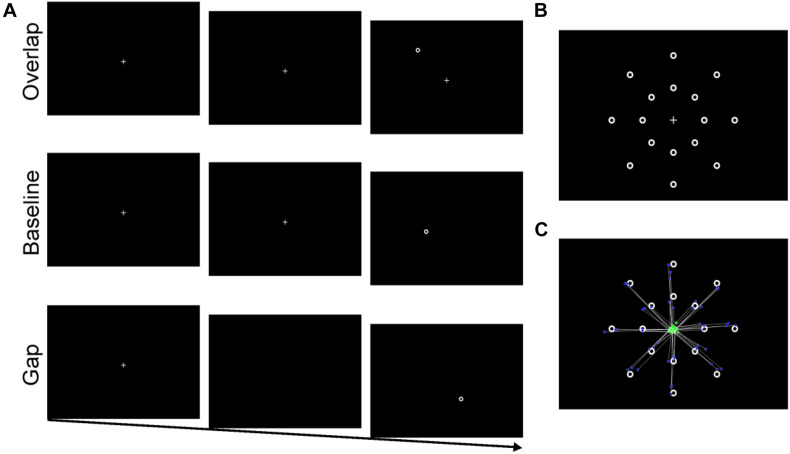
**(A)** Stimulus sequence for overlap, baseline, and gap conditions. **(B)** Stimulus array with 16 possible target locations (only one target was present for each trial). **(C)** An example of saccades from one participant (one block) included in the latency analysis. Green dots represent individual saccade start location and blue dots represent saccadic endpoint.

Eye-tracking equipment and participant setup were identical to the resting pupil paradigm. The central fixation was a crosshair (“+”) and the target was an annulus. At a viewing distance of 60 cm, the crosshair and annulus were approximately 1° by 1° visual angle. White fixation and target were displayed on a black background. There were 16 target locations arranged on two invisible concentric circles (8 per circle); circles surrounded the fixation cross at eccentricities of 4.9° (near) and 9.8° (far; see Figure 1B). For each trial, a single target was randomly presented in one of these locations.

#### Procedure

Participants first completed a nine-point calibration and validation procedure. As illustrated in Figure 1A, each trial began with the central crosshair presented alone for a random duration between 1000 and 1500 ms. Next, the peripheral target appeared either: (1) with the central fixation remaining onscreen (overlap condition), (2) 200 ms after the central fixation disappeared (gap condition), or (3) with the simultaneous offset of the central fixation (baseline condition). The target (and the central crosshair for overlap trials) remained onscreen for 2000 ms or until a target fixation had been made (minimum 200 ms). Then a 1000 ms inter-trial interval occurred during which only a blank black screen was presented. Prior to beginning the experiment, participants were instructed to look at the center fixation at the start of each trial, and then to move only their eyes to the target once it appeared. Participants completed a series of twelve practice trials prior to beginning the experiment.

#### Design

The experiment consisted of 144 trials, divided into three blocks of 48 trials. Within each block, condition (gap, baseline, and overlap), distance (near, far), and location (16 possible) were varied in pseudorandom order. All trial types were presented an equal number of times within each block and across the experiment.

#### Preprocessing and Analysis

To be considered a valid trial for subsequent analysis the following criteria had to be met: (1) at the onset of the target, participant’s gaze must be on the central fixation location, and (2) the endpoint of the initial saccade after target onset was located within approximately 2° of the target (anticipatory saccades [<100 ms] were removed; see Figure 1C). Saccadic reaction time (SRT) was defined as duration between the presentation of the peripheral target and the onset of the initial saccadic eye movement. No-shift trials required that no saccade was made within 2000 ms after target onset, and that fixation was maintained on the central crosshair. Percentage of no-shift trials was calculated as the number of no-shifts trials divided by the total number of usable trials. Finally, to simultaneously account for saccadic latency and no-shift percentage, disengagement efficiency was calculated [SRT/(1 – no-shift%)].

### Auditory Gap-Overlap Experiment

As noted above, methods and results from this experiment have previously been reported in Keehn et al. (2019). Thus, only an abbreviated description is reported below.

#### Apparatus and Stimuli

Participants were tested in a sound attenuated, darkened room, and seated comfortably approximately 1.5 m directly in front of five speakers (Hafler M5 Reference) positioned on stands at approximately eye-level. Speakers were positioned in a semi-circular array at 0° (i.e., directly in front of) and at 15° and 30° to the left and right of participant. Auditory fixation at the central location was a 500 Hz pure tone, and peripheral targets emitted from side speakers were white noise (similar to Shafiq et al., 1998). All stimuli were played at a comfortable listening level (approximately 60 dBA).

Saccadic eye movements were recorded using electrooculography (EOG) via a Biopac EOG100C amplifier at a sampling rate of 500 Hz. Two 4mm reusable Ag/AgCL shielded electrodes (Biopac EL254S) filled with conductive gel (5% NaCl, 0.85 molar NaCl) were applied at the lateral canthi of the left and right eye. Hardware gain was set to 5000 (corresponding to an input gain of ±2 mV), and filter bandwidth was set to 0.05–35Hz prior to digitization. Data were acquired using AcqKnowledge 4.3 software (Biopac Systems, Inc).

### Procedure

First, an EOG calibration procedure was completed. Participants were instructed to keep their head still and to move their eyes to each speaker, which were visible during calibration, when a sound was presented. No visual stimulus (e.g., a light) was presented in association with the sound. Prior to the start of the gap-overlap task, a black curtain was drawn in front of the speaker array approximately 1.2m from the participant, thus visually occluding speakers. Therefore, rather than fixate on a specific object (e.g., central crosshair in the visual gap-overlap task), participants fixated on a specific location (i.e., the source of the sound). Each trial began with a tone presented alone from the center speaker for a random duration between 1300 and 1500 ms. Next, a peripheral noise was played from one of the side speakers for 1200 ms either: (1) with the tone continuing to play for the duration of the peripheral noise from the center speaker (overlap condition), (2) 200 ms after the central tone stopped (gap condition), or (3) with the simultaneous offset of the central tone (baseline condition). Finally, there was a 2000 ms inter-stimulus interval during which time no sound was presented. Prior to beginning the experiment, participants were told they were going to play the “find the noise” game. They were instructed to look at the center location when the tone was playing, then to move only their eyes to location of the sound once the peripheral noise played, and then to look back toward the central location to wait for the tone. Finally, participants completed a series of six practice trials.

### Design

The experiment consisted of 108 trials, divided into three blocks of 36 trials. Within each block, condition (gap, baseline, and overlap), distance (near and far), and side (left and right) were varied in pseudorandom order. All trial types were presented an equal number of times within each block and across the experiment.

### Preprocessing and Analysis

Horizontal saccadic eye movements to the target locations were detected as abrupt changes in the EOG with a peak velocity greater than 50°/s for a duration of at least 20 ms for the duration of the peripheral noise (1200 ms). In addition, data from each trial were visually inspected by trained research assistants blind to group membership. To be included, initial saccadic eye movements were required to follow a steady fixation at the central location for at least 200 ms prior to target presentation. Trials in which there was no stable fixation at the central location (due to movements or noise) or trials in which the initial saccade was directed toward the incorrect side (e.g., saccade to left with target on right) were excluded. Saccadic reaction time (SRT) was defined as duration between the presentation of the peripheral noise and the onset of the first saccadic eye movement directed toward the side of the noise. Trials with SRTs less than 80 ms were considered anticipatory and excluded. Trials on which no saccade occurred but where fixation was maintained at the central location were coded as no-shift trials. Percentage of no-shift trials was calculated as the number of no-shifts trials divided by the total number of usable trials. Finally, to simultaneously account for saccadic latency and no-shift percentage, disengagement efficiency (DE) was calculated [SRT/(1 – no-shift%)].

## Results

As shown in Table 1, groups did not differ significantly on age, sex, or non-verbal IQ. Compared to the TD group, the ASD group did have significantly lower verbal IQ.

### Resting Pupil Dilation

**FIGURE 2:**
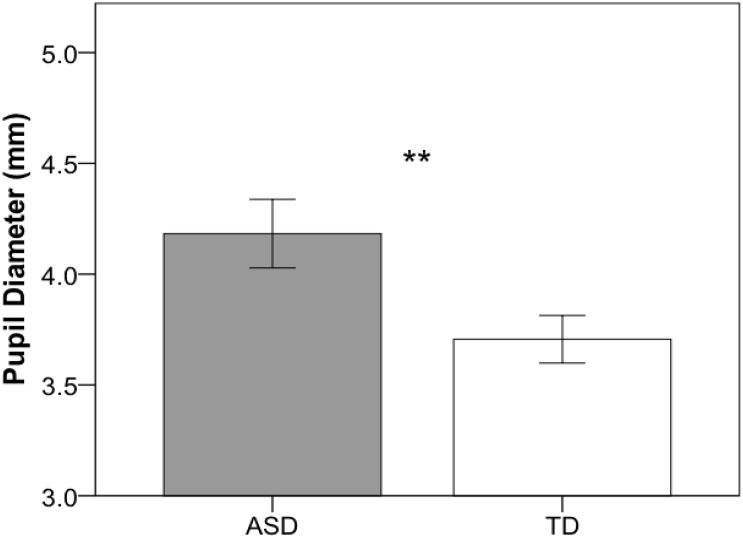
Mean pupil diameter for the resting eye-tracking task for ASD (gray) and TD (white) groups. Error bars represent ± 1 SEM. **p < 0.05.

Independent-samples *t*-tests were used to compare pupil diameter, head motion, and percentage of excluded data across groups. As illustrated in Figure 2, children with ASD (*M* = 4.18; *SD* = 0.71) showed significantly larger pupil diameter compared to their TD peers (*M* = 3.71; *SD* = 0.48), *t*(39) = 2.503, *p* = 0.017. Groups did not differ in head movement (ASD: *M* = 0.11; *SD* = 0.10; TD: *M* = 0.08; *SD* = 0.03), *t*(39) = 1.11, *p* = 0.275, but the ASD group had significantly more data excluded (ASD: *M* = 26.3%; *SD* = 9.9; TD: *M* = 10.1%; *SD* = 7.5), *t*(39) = 5.893, *p* < 0.001. However, percentage of data excluded was not associated with pupil diameter for either the ASD, *r*(20) = 0.084, *p* = 0.717, or the TD, *r*(19) = 0.362, *p* = 0.117, group. Additionally, to confirm that variability in age and IQ did not contribute to differences in pupil diameter across groups, separate ANCOVAs were conducted that included age and IQ as covariates. Children with ASD exhibited significantly larger resting pupil diameter when both age, *F*(1,38) = 7.58, *p* = 0.009, and non-verbal IQ, *F*(1,38) = 5.43, *p* = 0.025, were entered as covariates.

### Visual Gap-Overlap

Number of usable trials, saccadic reaction time (SRT), percentage of no-shift trials, and disengagement efficiency were analyzed using mixed-model repeated-measures ANOVA with between-subject factor group (ASD and TD) and within-subjects factors condition (gap, baseline, and overlap) and distance (near and far). In addition, the gap effect was calculated by subtracting SRT (or DE) for gap from overlap (overlap-gap) conditions and the step effect was calculated by subtracting SRT (or DE) for baseline from overlap (overlap-baseline) conditions for near, far, and combined target distances, and were analyzed using a repeated-measures ANOVA with between-subjects factor group (ASD and TD) and within-subjects factor distance (near and far).

**FIGURE 3:**
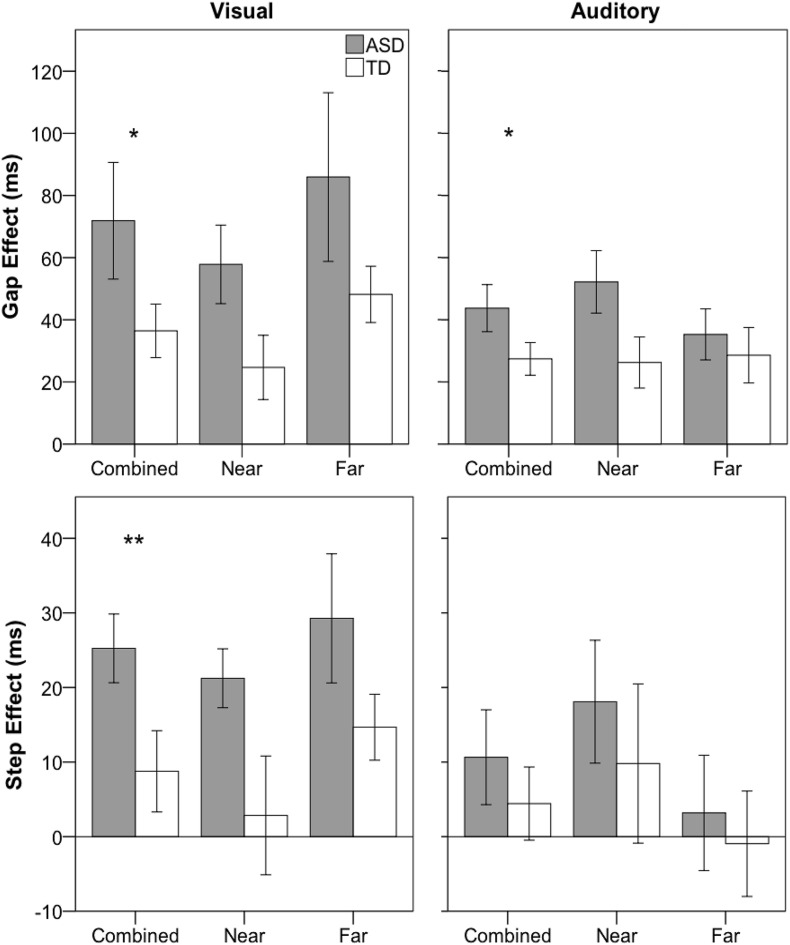
Saccadic reaction time gap (overlap-gap; top row) and step (overlap-baseline; bottom row) effects for visual **(left column)** and auditory **(right column)** for ASD (gray) and TD (white) groups. Error bars represent ± 1 SEM. **p < 0.05, *p < 0.1.

Children with ASD (*M* = 90; *SD* = 23) did provide fewer usable trials compared to TD children (*M* = 114; *SD* = 17), *F*(1,39) = 13.660, *p* = 0.001, η_*p*_^2^ = 0.26; however, there were no significant interactions between group and any factor for usable trials (all *p*-values > 0.4). For SRT, as expected, there were was a significant main effect of condition, *F*(2,78) = 22.295, *p* < 0.001, η_*p*_^2^ = 0.36 (gap < baseline < overlap; *p* < 0.001; gap: *M* = 158 ms; *SD* = 29; baseline: *M* = 175 ms; *SD* = 29; overlap: *M* = 213 ms; *SD* = 77). There was no main effect of group, *F*(1,39) = 1.111, *p* = 0.298, η_*p*_^2^ = 0.03, nor was there a significant interaction between group and any other factor (all *p*-values > 0.108). For the gap effect, children with ASD (*M* = 72 ms; *SD* = 86) showed a marginally significant increase compared to their TD peers (*M* = 36 ms; *SD* = 39), *F*(1,39) = 2.846, *p* = 0.0996; however, there was no significant interaction between group and distance, *F*(1,39) = 0.044, *p* = 0.834, η_*p*_^2^ = 0.00 (see Figure 3). For the step effect, there was a significant main effect of group (ASD: *M* = 25 ms; *SD* = 21; TD: *M* = 9 ms; *SD* = 24), *F*(1,39) = 5.381, *p* = 0.26, η_*p*_^2^ = 0.12, as the ASD group had a larger step effect compared to the TD group. There was no significant interaction between group and distance, *F*(1,39) = 0.099, *p* = 0.754, η_*p*_^2^ = 0.00.

No-shift percentage was non-normally distributed; thus, data were square-root transformed. For the percentage of no-shift trials, there was a significant main effect of condition, *F*(2,78) = 5.289, *p* = 0.007, η_*p*_^2^ = 0.12, as no-shift trials were most common in the overlap condition (gap < baseline < overlap; all *p*-values < 0.05). There was no main effect of group, *F*(1,39) = 0.837, *p* = 0.366, η_*p*_^2^ = 0.02, and no significant interaction between group and any factor (all *p*-values > 0.321).

Results for disengagement efficiency were similar; there was a main effect of condition, *F*(2,78) = 10.308, *p* < 0.001, η_*p*_^2^ = 0.21 (gap = baseline < overlap; *p* < 0.01), but no main effect of group, *F*(1,39) = 2.161, *p* = 0.150, η_*p*_^2^ = 0.05, or any significant interaction between group and any other factor (all *p*-values > 0.277). Finally, there were no significant main effects for disengagement efficiency gap or step effects (all *p*-values > 0.2).

### Auditory Gap-Overlap

Results have previously been reported in Keehn et al. (2019). Briefly, main findings involving group included: no main effect of group, *F*(1,39) < 1, nor were there any significant interactions between group and other experimental factors for number of usable trials (all *p*-values > 0.2), no significant main effect of group for SRT, *F*(1,39) < 1, and no significant interactions between group and any other factor (all *p*-values > 0.17). Similar to the visual paradigm, there was a marginally significant main effect of group for the gap effect (ASD: *M* = 44 ms; *SD* = 35; TD: *M* = 27; *SD* = 23), *F*(1,39) = 3.078, *p* = 0.087, η_*p*_^2^ = 0.03, but no significant interaction between group and distance, *F*(1,39) = 1.278, *p* = 0.265, η_*p*_^2^ = 0.03. Additionally, there was no significant main effect of group, or group by distance interaction for the step effect (all *p*-values > 0.4).

No-shift percentage was non-normally distributed; thus, data were square-root transformed. For percentage of no-shift trials, there was a significant interaction between group and condition, *F*(2,78) = 4.781, *p* = 0.011, η_*p*_^2^ = 0.11, and follow-up independent-samples *t-*tests showed that the ASD group had a significantly higher no-shift percentage for the overlap, *t*(39) = 2.336, *p* = 0.025, but not the gap, *t*(39) = 0.866, *p* = 0.392, or baseline condition, *t*(39) = −0.178, *p* = 0.860, compared to the TD group.

Significantly increased no-shift percentage and slower SRTs in the ASD group resulted in significantly decreased disengagement efficiency in the ASD group. Specifically, the group by condition interaction was significant, *F*(2,78) = 3.942, *p* = 0.023, η_*p*_^2^ = 0.09. For disengagement efficiency gap effect, there was a significant main effect of group, *F*(1,39) = 5.693, *p* = 0.022, η_*p*_^2^ = 0.12, with larger gap effects in the ASD (*M* = 53 ms; *SD* = 45) as compared to the TD (*M* = 27 ms; *SD* = 20) group; however, there was no significant interaction between group and distance, *F*(1,39) = 1.041, *p* = 0.314, η_*p*_^2^ = 0.03. For disengagement efficiency step effect, children with ASD showed marginally increased scores compared to TD children, *F*(1,39) = 3.916, *p* = 0.055, η_*p*_^2^ = 0.091, but no significant interaction between group and distance, *F*(1,39) = 0.795, *p* = 0.378, η_*p*_^2^ = 0.02.

### Associations Between Pupil Dilation and Disengagement Measures

**FIGURE 4:**
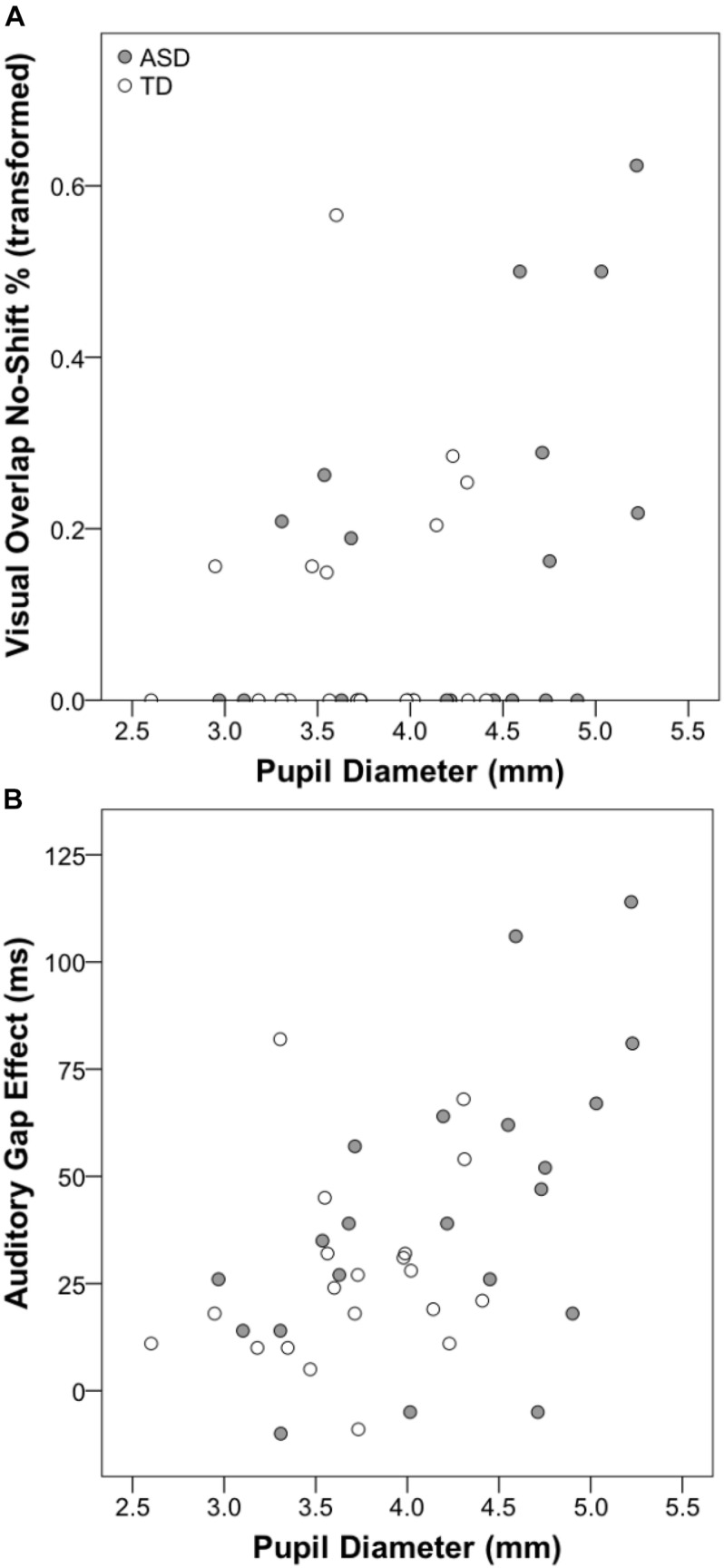
Scatterplots displaying associations between resting pupil diameter and visual gap-overlap task no-shift percentage **(A)** and auditory gap-overlap task gap effect score **(B)**.

Pearson’s correlations were used to investigate the association between resting pupil diameter and disengagement metrics (i.e., gap/step effects and percentage no-shift trials). For the visual gap-overlap experiment, across all participants there was a significant association between pupil diameter and overall percentage no-shift trials (see Table 2), which was due primarily to the association between pupil size and no-shift percentage for overlap, but not gap or baseline conditions. This pattern was present in the ASD group, but not the TD group (see Figure 4A). No other correlations were significant for the ASD group for the visual gap-overlap experiment.

**TABLE 2:** Visual gap-overlap correlations with pupil diameter.

		Gap effect			Step effect			% No-shift			
		All	Near	Far	All	Near	Far	All	Gap	Baseline	Overlap
All	SRT	0.130	0.058	0.256	−0.012	−0.062	0.102	0.330*	0.206	0.143	0.376*
	DE	0.232	0.164	0.212	0.264	0.211	0.338*				
ASD	SRT	0.198	0.116	0.264	−0.077	−0.284	0.089	0.419	0.237	0.277	0.464*
	DE	0.289	0.216	0.219	0.324	0.281	0.353				
TD	SRT	−0.374	−0.415	−0.066	−0.263	−0.195	−0.202	0.044	−0.136	0.001	0.111
	DE	−0.320	−0.454*	−0.001	−0.190	−0.429	0.102				

*p < 0.05, **p < 0.01; SRT, Saccadic reaction time; DE, disengagement efficiency.

For the auditory gap-overlap paradigm, there were significant correlations for all participants between pupil size and combined and near SRT and disengagement efficiency gap effects (see Table 3). Again, this pattern was present in the ASD but not the TD group. For the ASD group there were significant correlations between combined, near, and far SRT gap effects and combined and far disengagement efficiency gap effects (see Figure 4B).

**TABLE 3:** Auditory gap-overlap correlations with pupil diameter.

		Gap effect			Step effect			% No-shift			
		All	Near	Far	All	Near	Far	All	Gap	Baseline	Overlap
All	SRT	0.509**	0.373*	0.265	0.086	−0.087	0.509**	0.051	0.143	−0.179	0.126
	DE	0.533**	0.393*	0.304	0.309*	0.102	0.405**				
ASD	SRT	0.558**	0.479*	0.444*	0.196	0.083	0.243	0.120	0.233	−0.087	0.201
	DE	0.554**	0.387	0.484*	0.382	0.209	0.374				
TD	SRT	0.232	−0.061	−0.013	−0.116	−0.414	0.490*	−0.317	−0.137	−0.332	−0.619**
	DE	0.190	0.079	−0.222	−0.089	−0.394	0.408				

*p < 0.05, **p < 0.01; SRT, Saccadic reaction time; DE, disengagement efficiency.

## Discussion

The goals of the current study were to investigate whether tonic activation of the LC-NE system (as indexed by resting pupil diameter) is atypical in ASD, and to determine whether differences in attentional disengagement are associated with atypical tonic activity of the LC-NE system in children with ASD. In accord with previous reports, children with ASD exhibited significantly larger resting pupil diameter compared to their TD peers, which is indicative of increased tonic LC-NE activity. Furthermore, similar to prior findings, children with ASD showed subtle impairments in attentional disengagement in both visual and auditory gap-overlap tasks. Importantly, measures of disengagement were associated with our index of tonic LC-NE activity. Together, these results suggest that atypically increased tonic LC-NE activation may contribute to disengagement deficits in children with ASD.

First, our finding of significantly larger resting pupil diameter in ASD is in agreement with and extends earlier findings suggesting elevated tonic LC-NE activity in ASD (Anderson and Colombo, 2009; Anderson et al., 2013; Blaser et al., 2014; Top et al., 2018). Previous studies have shown larger pupil sizes in toddlers and young children (Anderson and Colombo, 2009; Anderson et al., 2013; Blaser et al., 2014) as well as adults with ASD (Top et al., 2018). The results of the present study extend these findings to older school-aged children and younger adolescents and suggest that atypically increased tonic LC activation may be present across the lifespan in individuals with ASD. Additionally, these findings are consistent with preliminary neuropharmacological research, which suggests that β-adrenergic antagonists (e.g., Propranolol), which block the action of norepinephrine, may improve functioning in a variety of behavioral domains in individuals with ASD (Beversdorf, 2020).

In addition to previously reported impairments in attentional disengagement for the auditory gap-overlap task (Keehn et al., 2019), results from the visual gap-overlap paradigm also suggest subtle deficits in visual attentional disengagement. Specifically, the gap effect was marginally increased and step effect scores were significantly larger in the ASD group. In both cases, these were due to disproportionally longer SRTs to overlap trials. However, contrary to our hypothesis, these group differences in disengagement did not vary based on the location (near or far) of the peripheral target. Prior findings of impaired disengagement from gap-overlap paradigms are mixed in ASD; however, our results add to the growing body of evidence that suggests that children with ASD exhibit impairments in disengaging attention.

The primary objective of the present report was to examine the associations between measures of tonic LC-NE activation and attentional disengagement. For both the visual and auditory gap-overlap tasks, the percentage of no-shift trials was significantly greater for overlap compared to other conditions, consistent with the premise that disengaging attention is more difficult when the central stimulus is present. For the visual task, we found that greater tonic activation of the LC-NE system was associated with increased overlap no-shift percentage for all participants; group-level analyses showed that this association was present for the ASD group, but not the TD group. For the auditory task, we found that greater tonic LC-NE activity was associated with both increased SRT and disengagement efficiency gap effect scores. Similarly, group-level analyses showed that this association was specific to the ASD group. ASD-specific correlations may be due to increased variability within the ASD group, which is likely associated with elevated resting pupil size and disengagement impairments in some, but not all, children with ASD. Furthermore, although the associations with specific disengagement indices varied across task, the direction of these relationships was consistent. For children with ASD, greater tonic LC-NE activation was associated with increased difficulty disengaging attention.

As highlighted previously (Forman et al., 1993), condition-specific changes in latency in gap-overlap paradigms are determined by more than one pathway. Much of the neurophysiological research investigating the gap effect has focused on the presence of faster SRTs and frequency of express saccades for the gap condition. However, in the current study and for the majority of previous findings, individuals with ASD show statistically equivalent and numerically faster SRT for gap trials. These findings suggest that the mechanism underlying group differences in disengagement may be unrelated to pathways responsible for gap-related changes in eye-movement dynamics.

Rather, findings from the present study suggest that atypically elevated tonic activation of the LC-NE system may contribute to poorer gap-overlap performance in children with ASD; although speculative, several interrelated theories of LC-NE function may explain this relationship. First, the adaptive gain model (Aston-Jones and Cohen, 2005) proposes that the LC operates in two modes: phasic and tonic. The phasic mode is associated with moderate levels of tonic activation, and reliable phasic responsivity to task-related stimuli, whereas the tonic mode is related to elevated levels of tonic activation with reduced phasic responsivity. Larger pupil dilation results from the resting eye-tracking task suggest that individuals with ASD may operate in the tonic LC-NE mode. Thus, one potential explanation for the association between atypically increased tonic LC-NE activation and disengagement impairments (increased overlap no-shift percentage and larger gap effects) is that, in the absence of a cue (fixation offset in gap trials), peripheral targets do not elicit significant phasic LC activation. Decreased or absent phasic LC activation to the onset of peripheral targets, due to elevated tonic LC activity, would likely result in increased saccade latency and/or more frequent no-shift trials. These findings are consistent with a recent report by Bast et al. (2021a), who showed altered LC-NE activity and slower reaction time in children and adolescents with ASD. These authors suggest that LC-NE tonic upregulation may decrease sensory selectivity contributing to increased reaction time latency in ASD.

The LC-NE system also plays a critical role in managing environmental uncertainty, acting as an interrupt signal in response to unexpected events (Yu and Dayan, 2005; Dayan and Yu, 2006). Similar to previous studies that have shown disengagement impairments in ASD, both visual and auditory gap-overlap tasks included variable duration for the fixation stimulus and randomized presentation of trial types (i.e., gap, baseline, and overlap presented within the same block). Prior research on neurotypical adults has shown larger switch costs associated with mixed (i.e., gap and overlap presented within the same block) compared to pure (i.e., just gap or overlap presented in each block) for SRT (Vernet et al., 2009). Moreover, the number of target locations used in the study tasks (16 for visual; 4 for auditory) is greater than most prior studies (typically two). As discussed previously (Keehn et al., 2019), these factors increase the unpredictable nature of the current paradigms and may contribute to disengagement differences, specifically slower SRTs and increased no-shift percentage for overlap trials, observed in the ASD group. According to Dayan and Yu (2006), changes in an individual’s task state in response to unexpected events are triggered by phasic NE responses, which act as an interrupt signals. Thus, similar to the adaptive gain model, elevated tonic LC-NE activity may disrupt phasic LC responsivity to unpredictable events (i.e., overlap trials; 33% of trials), resulting in disengagement differences in ASD.

Relatedly, in the current and previous gap-overlap tasks, participants were instructed to fixate the central stimulus and then to make an eye movement to the target once it appears; they were not explicitly told that fixation offsets cue impending targets. Nevertheless, participants learn the cue-target association and as a result SRTs to cued targets (i.e., gap and/or baseline conditions) are accelerated. However, overlap trials violate this antecedent-response expectation as overlap targets appear without fixation offset. Previous research in non-human primates has shown that atomoxetine, an NE-reuptake inhibitor that boosts the levels of NE, is associated with improved orienting in *predictive contexts*, but slower responses in non-cued conditions (Reynaud et al., 2019). These results suggest that NE may affect behavioral response patterns in a context-specific manner, speeding orienting to predictive cues (such as fixation offset in gap/baseline trials) and slowing reaction time to non-cued targets (such as in overlap trials). Furthermore, research by Gonzalez-Gadea et al. (2015) has shown reduced P3 amplitude, an indirect index of phasic LC-NE activation (Nieuwenhuis et al., 2005), to unexpected events in children with ASD. Additionally, findings from Goris et al. (2018) demonstrated that context-dependent modulations of the mis-match negativity (MMN) were also reduced in adults with ASD compared to the TD peers. Together, these results suggest that atypical responsivity to overlap trials in people with ASD may result from impairments in the ability to adjust precision when faced with uncertainty. In the context of the current study, strict application of a fixation offset – target onset expectancy may result in behavioral costs for conditions that violate that prediction, and this predictive processing may rely, in part, on LC-NE activation.

More recently research by Bast et al. (2021b) showed differences in oculomotor function in individuals with ASD, specifically decreased saccade duration and amplitude, which may be associated with reduced visual exploration. These authors hypothesized that the underlying mechanism associated with atypically clustered fixations may be altered pontocerebellar circuitry. Although the present study focused on attentional disengagement and not basic saccade dynamics, the LC does project to (oculo)motor neurons of the brainstem and cerebellum (Samuels and Szabadi, 2008), and may also contribute to atypical oculomotor/attentional function in individuals with ASD.

Locus coeruleus – norepinephrine disruption is not unique to ASD and has also been shown to be present in conditions such as ADHD (Del Campo et al., 2011) and anxiety (Morris et al., 2020), which are both frequently comorbid with ASD (Lai et al., 2019). Related to the present study, anxiety has been associated with impairments in attentional disengagement (e.g., from threat-related stimuli; Richards et al., 2014). Future research should use a transdiagnostic approach to examine the LC-NE system and attentional dysfunction in children with ASD, ADHD, anxiety, and other conditions, which may share overlapping genotypic and phenotypic features.

Finally, there are several limitations to the present study. Although groups were age-, IQ-, and sex-matched, they did differ on the percentage of usable data on resting pupil and visual disengagement paradigms. However, for resting pupil task, the amount of usable data was not associated with pupil diameter across all participants or within each group, suggesting that increased missing data in the ASD may not have contributed to larger pupil size in ASD. Additionally, our resting state pupil diameter analyses did not control for differences in gaze deviations from central fixation, and thus we cannot rule out whether systematic differences in fixation patterns across group contributed to the presence of pupil diameter differences. Lastly, although increased in the overlap relative to the gap and baseline conditions, no-shift trials were rare and did not occur frequently in participants. As such, correlations between tonic pupil size and no-shift percentage may have resulted from a small subgroup of participants with increased no-shift percentages.

## Conclusion

Difficulties in disengaging and shifting attention are present early and persist across the lifespan in individuals with ASD. These early fundamental differences in attention are associated with subsequent ASD diagnosis and may contribute to the emergence of the ASD phenotype. However, thus far, the neural mechanism(s) underlying impaired attentional disengagement in ASD remain unclear. Results from the present study confirm prior reports of larger resting pupil size in individuals with ASD, indicative of atypically increased tonic LC-NE activation. Furthermore, consistent with our hypothesis, we also found that individuals with ASD showed slower attentional disengagement, and differences in attentional disengagement in individuals with ASD were associated with elevated levels of tonic LC-NE activity. Together, these results suggest that atypical tonic activation of the LC-NE system is present in ASD and may contribute to difficulties in disengaging and orienting attention. Future research aimed at understanding the role of the LC-NE system in context-specific patterns of responsivity in ASD will further inform our understanding the neural bases of these attentional differences, and have the potential to contribute to the development of novel biobehavioral markers and behavioral and pharmacological intervention targets.

## Data Availability Statement

The raw data supporting the conclusions of this article will be made available by the authors, without undue reservation.

## Ethics Statement

The studies involving human participants were reviewed and approved by Purdue University Institutional Review Board. Written informed assent and consent to participate in this study were provided by all participants and participants’ legal guardian/next of kin.

## Author Contributions

BK conceived of and designed the study, performed the statistical analyses, and drafted the manuscript. AF participated in the design of the study. BK, GK, SB, and RM acquired the data. GK, RM, and AF helped to revise the manuscript. All authors read and approved the final version of the manuscript.

## Conflict of Interest

The authors declare that the research was conducted in the absence of any commercial or financial relationships that could be construed as a potential conflict of interest.

## Publisher’s Note

All claims expressed in this article are solely those of the authors and do not necessarily represent those of their affiliated organizations, or those of the publisher, the editors and the reviewers. Any product that may be evaluated in this article, or claim that may be made by its manufacturer, is not guaranteed or endorsed by the publisher.
